# Patients and Community Members as Equal Partners in Curriculum Decision‐Making

**DOI:** 10.1111/tct.70212

**Published:** 2025-09-21

**Authors:** Larry Leung, Jason Min, Kerry Wilbur

**Affiliations:** ^1^ Faculty of Pharmaceutical Sciences University of British Columbia Vancouver Canada

**Keywords:** community participation, curriculum, health professional education, Indigenous health, patient partners, pharmacy

## Abstract

**Background:**

Patients and community members make essential contributions to health professional education, which has consistently demonstrated a positive impact on student learning. However, patients and community members do not often have the opportunity for formal participation at higher levels outside instructional spaces. Teaching faculty leaders in the Faculty of Pharmaceutical Sciences at the University of British Columbia formed the Indigenous Advisory Committee (IAC) whereby community members contribute as equal partners in curriculum decision‐making.

**Approach:**

The IAC provided oversight and authority on the decolonisation and Indigenisation efforts in the pharmacy programme and was formally embedded in the governance model and reporting structure of the curriculum committee. Majority membership was Indigenous members to ensure these experts were the leading voice. The IAC developed five curricular pillars and accompanying core learning objectives to underpin all Indigenous content across the pharmacy programme and devised an engagement pathway for collaboration with community partners.

**Evaluation:**

Key changes were implemented in the pharmacy curriculum, including a mandatory Indigenous Health and Cultural Safety course, the first of its kind among pharmacy programmes in Canada. In formal committee feedback, community members described feeling valued as their input is reflected and prioritised in decision‐making. Student members were motivated to form a new social club for Indigenous students to connect and share cultural experiences.

**Impact:**

The work of IAC ensured relevant student courses and content was rooted in authentic lived experiences with a community‐informed focus on strengths and needs. Equal‐partner roles in curriculum decision‐making loosened conventional hierarchical structures and reinforced community relationships and forged new collaborations.

## 1 Background

Patients are essential to student training in health professional education programmes. Patient contributions to the curriculum, through sharing health experiences in the classroom, volunteering their stories as teaching cases and acting as standardised patients, have consistently demonstrated a positive impact on learning [1]. Students report enhanced communication and interpersonal skills, increased confidence in shared decision‐making, and improved empathy and patient‐centredness following these patient interactions [2]. These patient roles are familiar in health professional curricula, but other opportunities to integrate patients and community members into programming remain overlooked.

Towle and Godolphin are among the longstanding advocates for expanded and meaningful engagement of patient and community voices in health professional curriculum [3, 4]. They have proposed a spectrum of involvement, starting with important contributions such as: (1) patients' creation of learning materials; (2) standardised or volunteer patients; (3) patients sharing their health experiences, and extending to participation in (4) patients teaching and assessing students; (5) patients as equal partners; and (6) involvement in institutional decision‐making [5]. Unfortunately, 10 years have passed since this model was outlined, and advanced integration of patient and community members' roles in health professional education remains elusive. For example, recent reviews have found patient participation in health professional student workplace‐based assessment is currently lacking [6]. It is additionally rare to encounter patients or community members with formal institutional roles and associated responsibilities [7].

*It is rare to encounter patients or community members with formal institutional roles and associated responsibilities*.

The Faculty of Pharmaceutical Sciences at the University of British Columbia (UBC) is home to one of the largest undergraduate pharmacy programmes in Canada and is committed to delivering a contemporary curriculum responsive to societal needs. In 2015, the Truth and Reconciliation Commission of Canada's Report issued several Calls to Action to improve Indigenous health outcomes, address systemic inequities and ensure culturally safe healthcare services [8]. Accordingly, the University and our Faculty have prioritised decolonisation and anti‐racism in their strategic plans. Teaching faculty leaders in our programme are invested in collaborating with *patients as equal partners* in curriculum development and reform and have successfully incorporated patient and community members into educational decision‐making. We describe the development and work of an Indigenous Advisory Committee (IAC) to advance decolonisation and Indigenisation efforts in our pharmacy programme (Box 1).

Box 1 **Reflexivity statement.**

LL, JM and KW are non‐Indigenous health professional educators of Chinese, Korean and Anglo ancestry, respectively. LL and JM have provided direct patient care through medication management services in Indigenous communities since 2010, maintaining strong relationships with both urban and rural/remote Indigenous partners through their research lab, UPROOT. At the time of IAC formation, KW was the Executive Director responsible for the undergraduate pharmacy degree programme. We recognise the power and privilege we hold as clinicians, educators and researchers working in Indigenous health contexts. Our perspectives are shaped by our professional roles, lived experiences and the Western institutional frameworks we work within.

We approach this work with a commitment to relational accountability, cultural humility and respect for Indigenous knowledge systems. Throughout the inception and work of the IAC, the team reflected critically on positionalities and sought to ensure the work remained grounded in reciprocity and community relevance. The undergraduate pharmacy student research assistant (European ancestry) contributed meaningfully to this process, supported by mentorship and guidance from LL and JM. We continue to learn from the communities we serve and aim to contribute to equitable, respectful and strengths‐based Indigenous health education.

## 2 Approach

### 2.1 IAC

Formation and work of the IAC was part of a larger curriculum initiative. The IAC was formed in the first year of a 3‐year project, ‘UPROOT: A Community‐Based Approach to Decolonizing and Indigenizing the Pharmacy Curriculum’.

### 2.2 Membership

The 11‐member IAC was composed of Indigenous community partners (5), self‐identified Indigenous pharmacy students (2), non‐Indigenous healthcare professionals employed by Indigenous health service organisations (2) and two teaching faculty leaders. Representation included members from rural and underserved communities, as well as Indigenous Knowledge Keepers and Elders. The committee make‐up ensured Indigenous experts were the leading voice in committee conversations and decision‐making.

The teaching faculty leaders had built genuine and trusting relationships with community members through prior work as pharmacists in Indigenous communities [9]. Individuals accepting invitation to the IAC included Indigenous community partners who previously collaborated with these faculty leaders.

Community members of IAC were financially compensated for their time and expertise. In a further commitment to reciprocity, each member was invited to collaborate on a community project of their choice that would benefit their nation.

### 2.3 Mandate

The purpose of the IAC was to provide oversight and authority on the decolonisation and Indigenisation efforts in the pharmacy programme and ensure the prioritisation and respect of indigenous knowledge, values and practices. The committee approach was left undefined to ensure Indigenous members led conversations on how work would be conducted. The membership developed Terms of Reference ensuring full committee oversight on Indigenous curriculum decisions.

The IAC initially met quarterly and shifted bi‐annually thereafter. Committee meetings began with an Indigenous opening as a custom and sign of respect among many First Nations. Separate smaller meetings were conducted among members working on specific courses or aspects of the curriculum before additional discussion at IAC. Deliberations on strengthening, scaffolding and expanding Indigenous curricular content solicited all member perspectives, and decision‐making by consensus was sought. In addition to these regular meetings, an Indigenous gathering was hosted each year at the Faculty to celebrate the collaboration.

The IAC created five curricular pillars and accompanying core learning objectives to underpin all Indigenous content across the pharmacy programme: (1) colonialism; (2) power and privilege; (3) cultural safety and humility; 4) health and healing; and (5) ethical engagement (Table 1). The IAC members devised an engagement pathway for collaboration with community partners, including a standardised approach to identify, develop and approve Indigenous community‐based pharmacy project opportunities, including explicit roles and responsibilities of partners, students and faculty (Table 2).

**TABLE 1:** Five curriculum pillars underpinning Indigenous curriculum content.

Pillar	Description
Colonialism	Colonialism in Canada, past and present, involved systematic oppression of Indigenous peoples through land dispossession and cultural genocide. This included the residential school system and the 60's Scoop, which has led to lasting health disparities and social inequities. This pillar focuses on uprooting colonialism and the strength and resiliency of Indigenous peoples in regaining their Indigenous identity, governance systems and self‐determination.
Power and privilege	Power and privilege shape relationships, health and equity in society. Understanding Indigenous governance, systemic racism and personal positionality reveals how structures maintain inequality. This pillar focuses on challenging overt and covert racism and reflecting on one's role, especially in healthcare, as essential steps towards promoting justice and meaningful change.
Cultural safety and humility	Cultural safety and humility require healthcare providers to examine personal biases and cultural assumptions while understanding how trauma affects Indigenous patients. By understanding the impact of trauma and prioritising respectful, culturally‐safe approaches, healthcare providers can build trust and promote healing in diverse communities. This pillar emphasises the responsibility all members of the healthcare team have to create an environment that is safe for patients.
Health and healing	Health and healing encompass both Indigenous and Western knowledge systems, each offering distinct and complementary approaches to wellness. While operating within an evidence‐based Western approach to care, the awareness and respect for Indigenous traditional healing practices is an important reality with patients today. This pillar focuses on the complexity of acknowledging and operationalising Indigenous knowledge systems into the Western healthcare system to improve access to inclusive care.
Ethical engagement	Ethical engagement is a key component of allyship in the building respectful relationships with patients and Indigenous communities. Grounding engagement in the OCAP principles—Ownership, Control, Access and Possession, ensures that collaboration upholds community rights. This pillar focuses on strength‐based approaches in optimising community assets and supporting resiliency and self‐determination.

**TABLE 2:** Indigenous community‐based projects examples.

Community project titles	Summary description
Visual Summary of Traditional Medicines	The community identified a need to summarise traditional uses of local plants in a visual medium to be easily shared and displayed. This project created a visual summary of traditional medicines and local plants used by the health centre's local expert herbologist, in efforts to preserve and revitalise traditional medicines knowledge, including the incorporation of traditional language, pictures and descriptions of use.
Improving Palliative Care Services in a Rural BC First Nation	The community identified barriers making it difficult to optimise care in a respectful way for its palliative care population. This project aimed to bridge the gap between the different healthcare resources, including the local health department, health authority palliative care team, pharmacy and local practitioners.

*Note:* More project summary descriptions can be found at https://uproot.pharmsci.ubc.ca/projects/.

The IAC reported its decisions to the Entry‐to‐Practice Doctor of Pharmacy Program (Curriculum) Committee.

## 3 Evaluation

### 3.1 Indigenous Curriculum

Four key changes were implemented in the pharmacy curriculum as a result of the IAC: (1) 3 h of content introducing the importance of Indigenous health and cultural safety were developed and integrated into a first‐year course, Foundations of Pharmacy; (2) a new mandatory course, Indigenous Health and Cultural Safety, was created for second‐year pharmacy students, the first of its kind among pharmacy curricula in Canada; (3) a third‐year elective course, Pharmaceutical Care in Indigenous Health, was re‐developed to feature pharmacy students pairing with Indigenous community partners to work on a community‐based educational or research project; and (4) the IAC informed the appropriate scaffolding of our Faculty's pharmacy curriculum with UBC's Centre for Excellence in Indigenous Health's UBC 23 24 Indigenous Cultural Safety Program, which features interprofessional learning with Indigenous and non‐Indigenous facilitators.

### 3.2 IAC Member Feedback

An evaluation of the committee framework and associated processes for curriculum development included narrative inquiry of IAC member experiences gathered through one‐on‐one conversations [10]. All of the nine non‐faculty IAC members, including students, spoke to an independent research assistant (RA) who followed a semi‐structured topic guide. The audio recordings were transcribed and open coded by the RA. Topic summaries were then identified and deliberated by the RA with two faculty subject matter experts (LL, JM).

Member feedback was overwhelmingly positive. They valued the clear decision‐making authority to develop and implement curriculum change, as well as the committee's purposeful prioritisation of Indigenous ways of knowing and doing.

[The IAC is] a tremendous asset for looking at better ways of Indigenous inclusion and including perspectives.

any sort of committee or membership Indigenous‐wise is normally set up like a sharing circle […] with a talking stick … and it's been really appreciated to have everyone's full attention when you are talking about those vulnerable things.

*They valued the clear decision‐making authority to develop and implement curriculum change*.

While IAC members praised the atmosphere of comfort and safety set during meetings and gatherings, some expressed personal challenges balancing dual roles and questioned whether they were serving the committee by providing an Indigenous perspective or by sharing their general views as students or patients.

### 3.3 IAC Student Member Feedback

Similarly, student members felt unsure which input was more helpful for curriculum decision‐making: providing views as a learner or their own perspectives as community members. They also expressed some hesitancy in sharing perspectives in a group with prominent Indigenous leaders. Student member feedback prompted the creation of a separate Indigenous Student Advisory Subcommittee, allowing students to review and discuss agenda items with the teaching faculty leads prior to full IAC meetings. This Student Advisory Subcommittee laid the groundwork for the development and creation of a student‐led social club, the Indigenous Pharmacy Student Collegium.

## 4 Implications

The strategies and initiatives incorporating patients and community members into the development and delivery of healthcare services and research are well described, yet similar decision‐making roles in health professional education are limited [11, 12]. The creation of the IAC in our Faculty, rooted in principles of equal partnership and ethical engagement with community members, has yielded several benefits. Community members gain a sense of empowerment through contribution to curriculum decision‐making and a validating space to voice their experiences and perspectives. The formal position of the IAC in the governance structure of curriculum decision‐making, as well as IAC member compensation, further legitimises the importance of the partnership and recognised member contributions. The collaboration strengthens relationships between communities and the Faculty and expands opportunities for other partnerships.

*Community members gain a sense of empowerment through contribution to curriculum decision‐making and a validating space to voice their experiences and perspectives*.

The support of IAC ensures the teaching and learning of Indigenous health and cultural safety in our pharmacy curriculum is locally relevant and community‐informed. Student course content is represented in ways consistent with lived experiences of the communities in question. Students are encouraged to discuss, question and reflect to deepen understanding of complex health issues facing community members. Indigenous curriculum is often geared towards non‐Indigenous audiences, but the IAC also considered curriculum important for Indigenous pharmacy students, such as specific community‐based projects supporting practical learning. The unexpected, but welcomed, formation of the Indigenous Student Collegium provides the first formal forum for Indigenous students in our programme to connect and share cultural experiences. Faculty and staff exposure to the IAC and its recommendations helps loosen conventional hierarchical structures of curriculum decision‐making. Ultimately, a community‐informed Indigenous health curriculum design promotes student translation of knowledge, skills and attitudes into future patient healthcare encounters and a positive influence on culturally safe practice and policy in the healthcare system itself [2].

*A community‐informed Indigenous health curriculum design promotes student translation of knowledge, skills and attitudes into future patient healthcare encounters*.

Although the IAC's creation—and its intentional conduct for authentic engagement with Indigenous partners—addresses a specific educational mandate for decolonising and Indigenising health professional education in Canada, best practices for community engagement were enacted [13, 14, 15]. Such principles include institutional partners who are trustworthy, respectful, flexible and prioritise community ownership [7, 9]. The IAC as a model for equal partnership is adaptable to other contexts where formal engagement with community members for relevant course‐related consultation can expand perspectives on content (Box 2). We encourage health professional education programmes to pursue opportunities to integrate patients and community members into curriculum decision‐making.

Box 2 **Recommendations for integrating patient partners and community members in curriculum decision‐making.**

Establish clear roles and purposes:

- Define why community or patient members are being included
- Clarify roles (as decision‐makers vs. consultants, co‐teachers)
- Orient educators and community/patient members to clarify expectations

Prioritise representation and inclusion:

- Include diverse voices and avoid over‐reliance on few ‘professional patients’ or advisory members
- Partner with community organisations to recruit representative members and foster trust

Use co‐design or co‐creation approaches:

- Engage community partners early and throughout the curriculum development cycle
- Promote mutual learning and respect for different forms of expertise

Embed patient/community input into governance:

- Position member contributions into the systems of legitimate curriculum decision‐making to avoid tokenism
- Ensure decisions reflect community values, not just academic priorities

Compensate and support participation:

- Use consensus or deliberative processes where everyone's input is sought before moving forward
- Provide honouraria, transportation and technical support to enable equitable participation

Build respect based on shared goals and reciprocity:

- Incorporate relationship‐building time, such as check‐ins or shared meals
- Recognise member contributions in ways aligned with community values
- Support community‐identified needs for projects and training by ensuring curriculum deliverables have reciprocal benefits for partners.

Evaluate and reflect:

- Gather and use feedback to improve future engagement practices
- Regularly assess how community involvement impacts curricula and student learning

## Author Contributions

**Larry Leung:** conceptualisation, investigation, methodology, formal analysis, project administration, writing – review and editing, resources. **Jason Min:** conceptualisation, investigation, methodology, formal analysis, project administration, writing – review and editing, resources. **Kerry Wilbur:** writing – original draft, writing – review and editing, data curation. Dr. Leung and Dr. Min were faculty members of the IAC.

## Ethics Statement

Evaluation studies of the UPROOT Indigenous Advisory Committee were approved by the University of British Columbia Behavioural Research and Ethics Board #H20‐02415.

## Conflicts of Interest

The authors declare no conflicts of interest.

## Data Availability

The data that support the findings of this study are available from the corresponding author upon reasonable request.
